# Acute Severe Hyponatremia Induced by a Duloxetine Overdose in an Elderly Woman

**DOI:** 10.7759/cureus.10318

**Published:** 2020-09-08

**Authors:** Wesley D Oliver, Ryan D'Angelo, Jeffrey Gonzales, Tracey Wilson, Leah S Millstein

**Affiliations:** 1 Emergency Medicine, University of Maryland Medical Center, Baltimore, USA; 2 Cardiology, Thomas Jefferson University Hospital, Philadelphia, USA; 3 Internal Medicine: Critical Care, None, Baltimore, USA; 4 Medical Intensive Care Unit, University of Maryland Medical Center, Baltimore, USA; 5 Internal Medicine - Pediatrics, University of Maryland School of Medicine, Baltimore, USA

**Keywords:** duloxetine, hyponatremia, overdose, syndrome of inappropriate antidiuretic hormone secretion

## Abstract

We report a case of acute severe hyponatremia within 24 hours after a duloxetine overdose. An 82-year-old woman presented to the ED after ingesting duloxetine and diltiazem. She became hemodynamically unstable due to the diltiazem overdose and was appropriately resuscitated. During hospitalization she experienced hyponatremia consistent with syndrome of inappropriate antidiuretic hormone (SIADH). Based on the observations we concluded there was a probable relationship between the hyponatremia and the duloxetine overdose. Clinicians should monitor patients’ electrolytes for acute disturbances after an overdose of duloxetine.

## Introduction

Hyponatremia (serum sodium level <136 mmol/L) is a common electrolyte imbalance encountered in the hospital setting. Patients are often asymptomatic and resolution typically occurs with minimal intervention. Symptomatic hyponatremia, characterized by headache, vomiting, lethargy, altered mental status, necessitates more aggressive therapy to prevent seizure, coma, and death [1]. 

Hyponatremia is a well-documented adverse effect of chronic administration of selective serotonin reuptake inhibitors (SSRIs) and selective serotonin and norepinephrine reuptake inhibitors (SNRIs) [2-6]. The most likely cause of hyponatremia in these patients is the syndrome of inappropriate antidiuretic hormone (SIADH), which is characterized by euvolemia, low serum osmolality (<275 mOsm/kg of water), elevated urine osmolality (>100 mOsm/kg of water), and a urine sodium greater than 40 mmol/L. Treatment of SIADH-associated hyponatremia ranges from fluid restriction in asymptomatic patients to IV hypertonic saline in symptomatic patients [7-8].

There are multiple reports of patients developing hyponatremia upon initiation or while taking chronic duloxetine [7-18]. We present a case of acute severe hyponatremia due to an overdose of duloxetine, which has not been previously described. 

## Case presentation

An 82-year-old Caucasian woman, weighing 70.5 kg and 160 cm tall, being treated with duloxetine for depression presented to the ED within one hour of ingesting approximately 15 duloxetine 30 mg capsules (450 mg) and the same number of extended-release diltiazem 120 mg tablets. The patient became hemodynamically unstable in the ED due to the quantity of diltiazem prompting treatment with activated charcoal, calcium gluconate, normal saline, intralipids, and high-dose insulin euglycemic therapy. During this period of time the patient received two liters of normal saline, one liter of dextrose 10%, and one liter of dextrose 20% as part of the high-dose insulin euglycemic therapy. The patient's hemodynamic instability due to the diltiazem overdose was appropriately treated.

The patient’s medical history was significant for depression, hypertension, and hyperlipidemia. She reported previous allergic reactions to sulfa with an unknown manifestation. Home medications included duloxetine 30 mg orally daily, diltiazem extended-release 120 mg orally daily, and atorvastatin 40 mg orally daily. During the two months prior to this incident, the patient was taking duloxetine and had two sodium results of 138 and 140 mmol/L, which were presumed to be her baseline levels. It is unknown how long the patient had been taking duloxetine prior to this two month period.

The patient’s admission sodium level was 133 mmol/L (normal range: 136-145 mmol/L) and serum osmolality was 280 mOsm/kg (normal range: 275-295 mOsm/kg) [7-8]. During day 2 of hospitalization, the serum sodium decreased to 122 mmol/L. The hyponatremia was conservatively managed with fluid restriction of 1,000 mL/day and diuresis. In an effort to continue management of her psychiatric disease, the patient received a dose of duloxetine on hospital day 3 and day 4. During day 4, the serum sodium reached a nadir of 118 mmol/L. 

**Figure 1:**
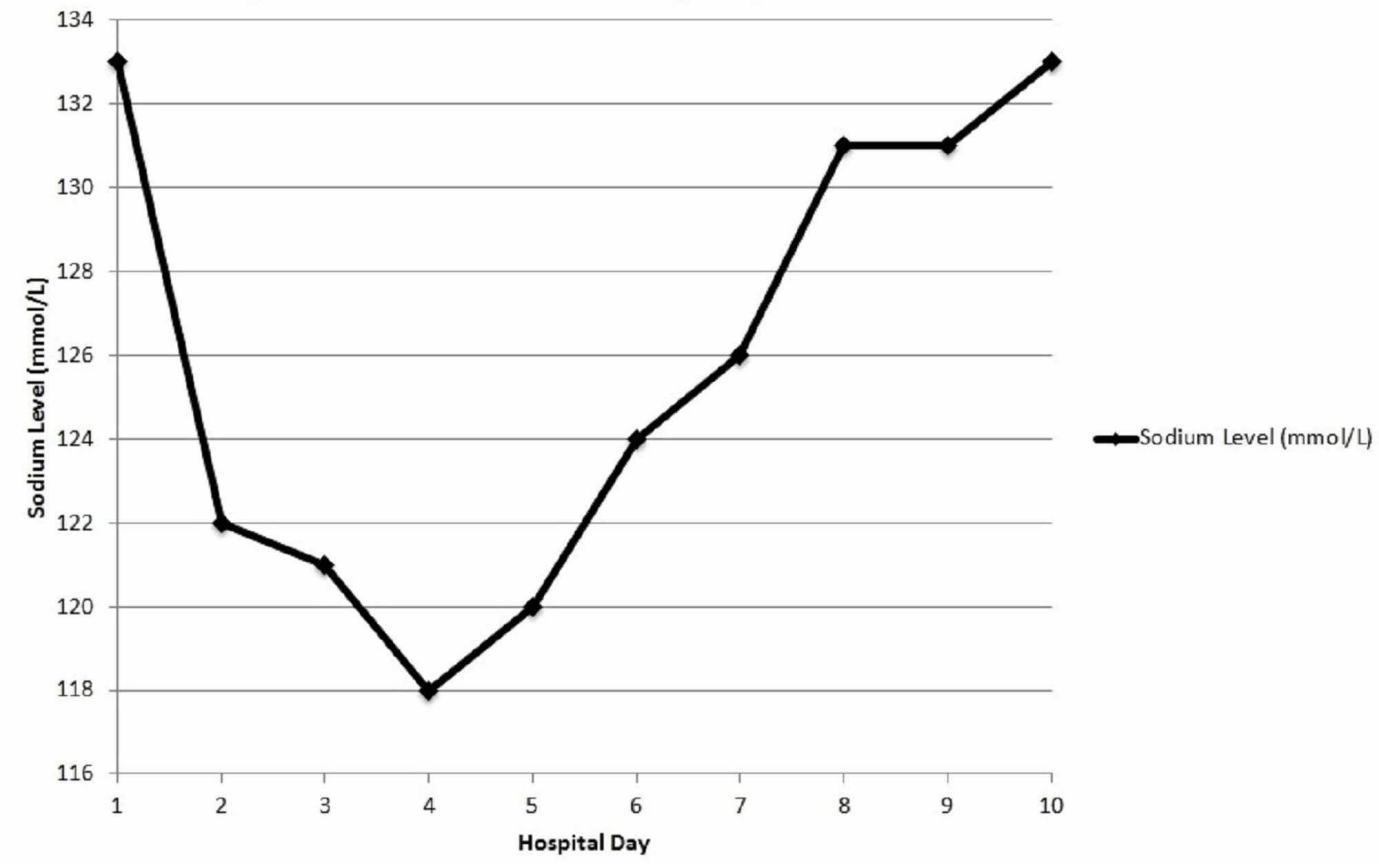
Sodium levels during hospital admission. Note: The patient had a baseline sodium of 140 mmol/L approximately two months before admission.

On day 5 of hospitalization, urine tests revealed an increased urine osmolality of 354 mOsm/kg (diagnostic if >100 mOsm/kg) and an increased urine sodium of 113 mmol/L (diagnostic if >40 mmol/L), consistent with SIADH [7-8]. The patient’s sodium slowly increased to 133 mmol/L on day 10. See Figure 1 for the trend of sodium levels during hospital admission.

Alternate etiologies of hyponatremia, such as adrenal, thyroid, and renal disease, were excluded during this admission. The patient was ultimately transferred to a psychiatric floor after being medically cleared and subsequently discharged home.

## Discussion

Our patient developed hyponatremia within 24 hours of ingesting a large amount (estimated 450 mg) of duloxetine and diltiazem. To our knowledge, this is the first documented report of acute severe hyponatremia in the setting of a duloxetine overdose. 

There are many potential causes for hyponatremia in the elderly population; thus, alternate etiologies, such as adrenal, thyroid, and renal disease, were excluded during this admission. No other risk factors for hyponatremia in this patient were identified. Use of the Naranjo Adverse Drug Reaction Probability Scale indicated a probable relationship between the adverse effect of hyponatremia and the duloxetine overdose in this patient, which is consistent with our conclusion that duloxetine is the likely cause of hyponatremia [19].

Duloxetine-induced hyponatremia is thought to be caused by the induction of SIADH. It has been suggested that both norepinephrine and serotonin can stimulate antidiuretic hormone (ADH) secretion and duloxetine inhibits the reuptake of both; however, the exact mechanism of duloxetine-induced SIADH has not been elucidated [2, 13].

Duloxetine exhibits a half-life of up to 17 hours, thus resolution of hyponatremia could be expected over four to five days [20]. Our patient reached a nadir of 118 mmol/L on day 4 of hospitalization; however, administration of additional duloxetine doses on days 3 and 4 of hospitalization and the large overdose of duloxetine (450 mg) prior to admission may have prolonged stabilization to seven days. Previous reports have documented hyponatremia within two to five days after initiating duloxetine or after a dose escalation on chronic duloxetine therapy, but have not demonstrated a case this severe and abrupt in the setting of an overdose [7, 9-18]. 

Amoako et al. described a 76-year-old female treated with duloxetine for fibromyalgia. The patient’s serum sodium decreased to 118 mmol/L approximately six days after admission and she was diagnosed with SIADH. After discontinuing duloxetine, the patient’s serum sodium increased to 130 mmol/L after three days [7].

Choi et al. described a 58-year-old male being treated with duloxetine for somatic and depressive symptoms. The patient was started on duloxetine 30 mg daily, which was increased to 60 mg daily five days after initiation. The patient’s initial sodium was 135.1 mmol/L; however, six days after initiation of duloxetine the serum sodium decreased to 122 mmol/L. The patient was subsequently diagnosed with SIADH and duloxetine was discontinued. The serum sodium level normalized to 135.4 mmol/L two days after discontinuation [9].

Hu and Wurster reported an 81-year-old female who was started on duloxetine 30 mg daily for mood stabilization. The patient developed hyponatremia with a sodium of 120 mmol/L after two doses of duloxetine. The patient’s baseline sodium level was 131 mmol/L. The patient was diagnosed with SIADH, the medication was discontinued by the ED physician, and the patient was instructed to follow-up with the outpatient provider [10].

Kulkarni described a 38-year-old female who was started on duloxetine for generalized anxiety disorder. After four days of therapy she was found to have a serum sodium of 117 mmol/L. Duloxetine was stopped and her serum sodium improved to 138 mmol/L after five days. Two weeks later duloxetine was restarted and her serum sodium was 124 mmol/L after two days. Serum sodium levels returned to normal after duloxetine was discontinued the second time [12].

Mori et al. reported an 86-year-old female who was started on duloxetine 20 mg daily and trichlormethiazide 2 mg daily six days prior to hospital admission. Upon admission she was found to have a serum sodium level of 116 mmol/L and she was diagnosed with SIADH. The medications were discontinued and she was treated with fluid restriction, diuretics, and oral sodium chloride. Serum sodium increased to 123 mmol/L on the third day of admission and further increased to 137 mmol/L on the seventh day of admission, after which therapies were stopped [13].

Sun et al. presented a case of a 68-year-old male with major depressive disorder who developed hyponatremia one month after initiating duloxetine 30 mg daily. The patient was admitted to the hospital with complaints of unsteady gait, dizziness, nausea, malaise, decreased oral intake, and insomnia. His initial sodium of 130 mmol/L subsequently decreased to 127 mmol/L on day six of the hospital admission. The patient’s baseline sodium was noted to be 137 mmol/L. The patient was diagnosed with SIADH and duloxetine cross-titrated to escitalopram. The patient’s hyponatremia resolved approximately eight days after discontinuing duloxetine [15].

Wang et al. reported a 78-year-old who developed hyponatremia and delirium after taking two doses of duloxetine 60 mg daily for postherpetic neuralgia. The initial sodium was 125 mmol/L. Duloxetine was discontinued and within one week the patient’s sodium returned to normal [16].

Yang and Wu described a 57-year-old female with major depressive disorder treated with duloxetine. The patient was initiated on duloxetine 30 mg daily and after three weeks with no improvement in symptoms the dose was increased to 60 mg daily. Three days after the dose escalation the patient was found to have a serum sodium of 114 mmol/L and was diagnosed with SIADH. Duloxetine was discontinued and the patient was treated with fluid restriction, hypertonic saline, and diuretics. The serum sodium increased to 130 mmol/L after three days [17].

Yoshida et al. described a 77-year-old female who developed hyponatremia after a single dose of duloxetine. Upon admission her sodium was 119 mmol/L, with her baseline being 135 mmol/L one day prior. The hyponatremia resolved after two days [18]. 

A potential contributing factor to our patient’s hyponatremia could have been the fluids used during the first 24 hours. These fluids were used during high-dose insulin euglycemic therapy for the diltiazem overdose and there are no documented reports of hyponatremia or development of SIADH during this therapy; thus, it is unlikely that the administration of these fluids contributed to the observed hyponatremia.

## Conclusions

We report the first documented case of acute severe hyponatremia from an overdose of duloxetine in a patient previously stable on an appropriate chronic dose. Given the time of onset of hyponatremia, diagnosis of SIADH, and time course to resolution, it is probable that the overdose of duloxetine is the cause of hyponatremia in this patient. Given the nature of this case, clinicians should monitor patients’ electrolytes for acute disturbances after an overdose of duloxetine.
